# Impact of pre-exposure and post-exposure prophylaxes prevention programme on HIV burden and services in a low-resource setting: a simulation modelling approach

**DOI:** 10.11604/pamj.2021.40.163.26486

**Published:** 2021-11-17

**Authors:** Adekunle Olatayo Adeoti, Eren Demir, Shola Adeyemi, Usame Yakutcan, Andre Pascal Kengne, Gbenga Kayode, Ahmad Aliyu, Nneoma Idika, Christian Isichei

**Affiliations:** 1 Department of Medicine, Ekiti State University/Ekiti State University Teaching Hospital, Ado-Ekiti, Nigeria,; 2 University of Hertfordshire, Hertfordshire Business School, AL10 9AB, Hatfield, United Kingdom,; 3 Statsxperts Consulting Ltd and Bohemian Smartlytics Ltd, Haverhill, CB9 8PP, United Kingdom,; 4 South African Medical Research Council, Francie van Zijl Drive, Parow Valley, Cape Town, Western Cape, South Africa,; 5 Institute of Human Virology, Nigeria, Maina Court, Herbert Macaulay Way, Central Business District, Abuja, Nigeria,; 6 Institute of Human Virology, University of Maryland School of Medicine, Baltimore, USA,; 7 Faith Alive Foundation-Nigeria, Jos, Nigeria,; 8 University of Jos, Jos University Teaching Hospital, Department of Chemical Pathology, Jos, Nigeria

**Keywords:** Prevention, PrEP/PEP, HIV/AIDS, simulation, patient flow, service utilisation

## Abstract

**Introduction:**

sub-Saharan African countries contribute substantially to the global HIV disease burden. Despite this burden, and the promises that prevention could deliver, the implementation and uptake of HIV prevention programmes are still low. The study used the decision support system model to explore the potential impacts of prevention implementation on HIV burden (incidence) and service delivery.

**Methods:**

an operational research technique known as discrete event simulation model was used to capture an individual patient´s pathways through the HIV care process from diagnosis to treatment and monitoring. The regular monitoring, over a 5-year period, including all the activities and resources utilized at each stage of the pathway were analysed, and the impact of increasing prevention measures for an HIV treatment service in a treatment centre in Nigeria was tested using the simulation model.

**Results:**

forty-three patients currently access the Pre-Exposure Prophylaxis (PrEP) and Post Exposure Prophylaxis (PEP) annually, with a 20% and 80% split in the number of patients offered PrEP and PEP, respectively. Scenarios-based on increasing the number of people offered PrEP and PEP from 43 to 250 with a 50/50 split were tested. The outputs revealed improved preventive care by averting new HIV cases, reduction in service demand and utilization, but an increase in the required human resource as well as financial burden. In the next 5 years, the cumulative averted HIV cases are expected to increase from 2 and 5 people (baseline) to 24 and 20 people for PrEP and PEP, respectively. The potentially averted 2 cases per infected persons based on the basic reproductive number of HIV.

**Conclusion:**

the effective implementation of PrEP/PEP programme offers an additional safety measure to prevent HIV transmission in at-risk individuals and possibility of ending HIV epidemic.

## Introduction

Globally, HIV/AIDS is still a major public health challenge. In 2019, the World Health Organization reported 690 000 deaths from HIV-related causes and 1.7 million newly infected people living with HIV(PLWH) [1, 2]. The emergence of new pharmacological prevention strategies in HIV transmission has further enhanced the optimism in the effective control of HIV/AIDS with the aim of elimination and ending the epidemic. Pre-exposure and post-exposure prophylaxes (PrEP/PEP) are crucial to this successful implementation of preventive strategies in HIV control programme. The effective and consistent use of PrEP/PEP offers an additional safety measure to prevent disease transmission in at-risk individuals [3].

The introduction of PEP in the 90s, as a 28-day preventive course for both occupational and non-occupational exposure to HIV and the more recent complement with PrEP have significantly reduced HIV transmission, even in high-risk contacts [4]. Their efficacy, however, depends on drug adherence and retention in care within the period of treatment [5]. Despite this innovation, there are potential challenges in the patients´ flow that could hinder the effectiveness of this programme such as long waiting times, staff shortages, poor clinic organization, and laboratory delays [6]. Hence, the need for an urgent development of an in-built system for the assessment of the practice and service utilization by the stakeholders like clinicians, managers, and trialist which is unique in addressing the patients´ interest within the clinical setting, towards achieving improved patients´ adherence to treatment and better service delivery.

Nigeria is one of the high burden countries, with an estimated 1.9 million PLWH [1]. About half of the new HIV infections in Nigeria were due to low-risk sexual behaviours and mainly seen in serodiscordant couples [7]. Hence, the targeted preventive intervention like PrEP/PEP would strategically reduce HIV transmission. While several studies have investigated the awareness and determinants of PrEP/PEP utilization, despite the low level of PrEP/PEP awareness in this key population [8, 9] little is known about the impact of PrEP/PEP on HIV burden and service delivery. For instance, the number of averted cases, the available resources in the facility and the projected impact on the course of the disease over a period of five or ten years were not demonstrated in such studies.

When assessing the impact of PrEP and PEP on HIV burden and service utilization, developing traditional cost-effectiveness analysis or a statistical model is insufficient [10, 11]. These types of models would only enable the estimation of the averted patients' and determination of cost-effectiveness of the intervention [12]. Focusing on just these metrics does not tell the full story for practitioners to make an informed decision. For instance, increasing the use of PrEP/PEP for those at risk of HIV would result in an increase in the number of visits. This would have a knock-on effect on staff workload and the service needs to ensure the necessary resources are in place.

Therefore, a comprehensive view of an entire patients´ pathway within an HIV service is needed to capture all the uncertainties and variations. This will enable key decision-makers to assess the impact of change in greater detail, not just on one aspect of the service (e.g. the averted number of patients), but the entire service (activity, utilization of resources, staff requirements, budgeting and financial planning). We, therefore, developed Smart HIV Manager, an innovative technology in HIV disease management. If utilized and complied with, would integrate the care providers, HIV managers and their patients into one platform with the aim of providing better care for PLWH. Our study evaluates the impact of PrEP/PEP using a novel artificial intelligence (AI) technology, assessing the impact on HIV burden (incidence) and service utilization in a Nigerian HIV treatment centre.

## Methods

A simulation modelling approach of a statistically validated discrete event simulation (DES) was developed to assist clinicians, service managers and policymakers in decision-making. The selection of the methodology depends on the problem context and the system of interest. Where systems and processes are complex, analytical techniques are limited in use, whereas simulation, in particular DES, has the capability of modelling very complex systems like HIV disease [13].

DES is a technique used to depict a system within a computer simulation environment to observe its behaviour and state changes over time [14]. It is a well-established approach by the healthcare community, for its adaptability, suitability, and scalability. We developed a DES model capturing individual patient´s movements from the initial diagnosis to treatment and monitoring over 3, 5 and 10-year period (including all the activities and resources utilized at each visit like diagnostic activities, ART treatment, disease progression, counselling and intensified adherence counselling).

The conceptualization process was carried out with services at several countries including Nigeria, United Kingdom, South Africa, and Kenya. The team is made up of HIV physicians, HIV service managers, policymakers and HIV researchers. Therefore, the pathway was captured to a sufficient level of detail such that it is applicable to a wide range of HIV services around the world. The World Health Organization (WHO) guidelines [15] formed the baseline pathway, where necessary adjustments were made to ensure individual service provision of countries was considered.

There are five stages that patients typically go through within an HIV service around the world: prevention, diagnosis, treatment, monitoring and disease progression. At each stage of the pathway, resources are attached, such as a physician, nurse, lab technician, a room, and a counsellor. Further details about each patient attribute is also captured, e.g. their age group, ART regimen, viral load and CD4 count, pregnancy status, sex, etc. Depending on the patient´s type and attribute, frequency of visits, comorbidities, HIV suppressed/failed, clinical outcomes and adherence/non-adherence are modelled accordingly. By capturing this level of detail, we can depict real-life HIV care to a certain degree of accuracy within a computer simulation environment.

To test and validate the model, a data template was created to collect the input parameters from three different HIV centres, two in Nigeria and one in Kenya. Sub-Saharan Africa has the highest burden of HIV/AIDS, hence centres in west and East Africa were selected based on the number of patients regularly seen in clinics and the willingness to participate in the study. The two centres in Nigeria were further selected based on the geopolitical distribution where one centre from the northern (Faith Alive Foundation, Jos) and the other from the southern (Ekiti State University Teaching Hospital, Ado-Ekiti) region of Nigeria were included in the study. The Kenya Medical Research Institute in Kisumu and Siaya counties also participated in the validation process of the model. A total of 93 input parameters were established covering the entire HIV pathway of care. Where data was not available, we resorted to the literature, expert opinions and online resources. Country-level HIV treatment costs for “Budgeting and Financial Planning” purposes were collected through an exhaustive review of the literature covering 139 countries globally. Costing data were collected in US dollars or Euro for each country, which included first-line/second-line ART costs per person per year, laboratory cost per person per year, and overhead and personnel cost per person per year. Overhead costs included facility utilities (water, electricity, fuel and maintenance of generator), facility support staff (guards, cleaners), facility-level administrative staff, general consumables/other supplies at the site, transport/monthly running costs of vehicles (if used for ART).

### Operational definitions

**Prevention:** measured by the utilization of HIV prevention programme in the HIV treatment centre with the associated outcomes following the PrEP/PEP administration (e.g. number of averted HIV cases, cost-effectiveness analysis, number of visits, etc.).

**Service demand and utilization:** measured using the respondent´s access and utilization of HIV care services in the centre evaluated by examining the impact of the prevention programme generating key outputs, such as the difference in the number of PLWH due to prevention, visits broken down by patient type (naïve, treatment experienced, PrEP/PEP) and age group etc.

**Human resource planning and management:** outputs are established using the WHO Workload Indicators of Staffing Need (WISN). This method is implemented within the system to calculate available hours dedicated for treating PLWH by each staff type and the required numbers, i.e., doctors, medical laboratory scientists, HIV specialist nurses, dieticians, general nurses, and counsellors.

**Budgeting and financial planning:** measured by estimating the cost of treating PLWH and related patients in prevention programmes for each fiscal year.

The simulation model is a web-based platform with dashboards developed using Java Spring boot, Spring Data, Spring Security and Thymeleaf. An exhaustive set of easy to understand visualization are generated, forecasting over 3, 5 and 10-year period, covering key areas of concern in the management of HIV, generating a wide range of dashboard outputs, such as prevention, UNAIDS, budgeting, resource planning, and monitoring and evaluation.

As the simulation runs, the front interface is animated, enabling users to interact and communicate, thus providing the opportunity to observe the behaviour of their system under various conditions. For example, patients do not only flow through instantaneously, but also spend time and consume healthcare resources. DES provides insights on the cause -and- effect relationship between demand and capacity. As a result, patients might wait if the resource is not available at that moment, which can be seen in the animation.

Verification and validation of the model are critical for accountability and to ensure the results are robust, reliable and accurate. This is a vital process in any simulation model building, where all stakeholders, made up of clinicians, service managers and nurses were engaged in every step of the development. [15, 16]. The simulation outputs were compared with real-world outputs, which were 5% either side of the expected result. This suggests that the model accuracy is ~95% and is fit for purpose.

## Results

The impact of increasing prevention measures for an HIV treatment service in Nigeria was tested via the developed simulation model. At present, the service provides Pre-Exposure Prophylaxis (PrEP) and Post Exposure Prophylaxis (PEP) to around 43 patients annually. There is a 20% and 80% split in the number of patients offered PrEP and PEP, respectively. The selected scenario is based on increasing the number of people offered PrEP and PEP from 43 to 250 with a 50/50 split. A wide range of outputs was generated via the model, e.g. demand, activity (monitoring visits, counselling), staff hours, costing, and the number of averted cases due to prevention. The key findings are presented in the subsections below, namely i) Prevention ii) Service Demand and Utilization iii) Human Resource Planning and Management, and iv) Budgeting and Financial Planning.

**Figure 1:**
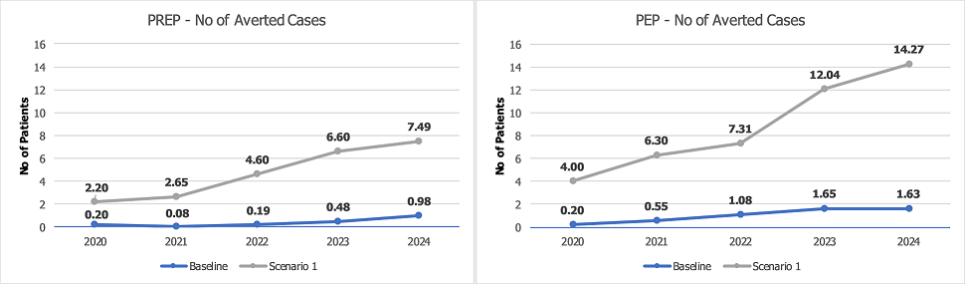
number of averted cases based on PrEP/PEP administration respectively

**Prevention:** the number of monitoring visits due to prevention is expected to increase dramatically. For instance, the number of visits during the 5-year period is expected to increase from 510 and 564 visits (baseline) to 7,681 and 2,062 visits for PrEP and PEP, respectively (Table 1). PrEP visits increased sharply due to the following reasons. Firstly, the proportion of people offered PrEP increased from 20% (baseline) to 50%. Secondly, patients on PrEP programme are not usually discharged unless they drop out (around 5%). Therefore, the number of patients in the PrEP programme cumulatively increases, whereas patients on PEP visits the service three times (within 6 months) and then discharged. The number of averted HIV cases due to PrEP/PEP remarkably increased throughout the years. The cumulative numbers (over 5 years) increased from approximately 2 and 5 people (baseline) to 24 and 20 people for PrEP and PEP, respectively. The number of averted cases in each year are illustrated in Figure 1.

**Table 1:** total number of visits during the 5-year period (from 2020-2025) and number of people on HIV treatment and prevention by 2025

Number of Monitoring Visits	Baseline	Scenario 1
PrEP (Prevention)	510	7,681
PEP (Prevention)	564	2,062
**Total**	**1074**	**9,743**
**Number of People by August 2025**	**Baseline**	**Scenario 1**
Treatment Naive	119	111
Treatment Experienced	1327	1319
**TOTAL PLWH**	**1,446**	**1,430**
PrEP	44	627
PEP	41	156
**TOTAL (Prevention)**	**85**	**782**

**Service demand and utilisation:** the impact of the intervention on service demand and utilisation was evaluated by examining the difference in the number of PLWH and prevention programmes (Table 1). The number of PLWH on treatment decreased to 1,430 (Scenario 1) from 1,446 (baseline). This shows that the increase in prevention measures (from 43 to 250 people per year) would make a positive impact on the programme by reducing new HIV cases and resource utilization that would have been needed for averted HIV cases. On the other hand, a noticeable reduction is expected in the number of monitoring visits by PLWH, i.e. from 20,711 visits over the 5 years (Baseline) to 20,593 (Scenario 1). This is due to the number of averted cases (i.e. Naïve patients). The number of monitoring visits by patient type and resource needs are presented for each year in Annex 1 the Appendix file.

**Human resource and management:** over the 5-year projection, “Total Hours” spent by all staff types increased from 32,608 hours to 40,231 hours due to the demand increase in PrEP/PEP. Specifically, the number of service hours for doctors increased by 20%, from 13,104 hours to 15,530 hours in total (see Annex 1 in the appendix). The number of available, required, and optimal staff for each staff types for the first year are presented in Table 2. The service currently has 5 doctors, and if they were to treat patients based on 100% utilisation rate (after excluding all WISN factors and the fact that 50% of their time are allocated for HIV patients only), then 4 doctors are required to cope with the current demand for HIV care. Note that this is the current practice (i.e. baseline) and does not assess the impact of any scenario. However, 100% utilisation rate is not a realistic assumption and based on a globally accepted utilisation rate of 80%, the optimal number of required doctors is 5. In the case of the scenario of interest (increasing PrEP/PEP from 43 to 250 people per year), the service needs minimum 5 doctors (100% utilisation rate) and 6 doctors as optimal (80% utilisation). In addition, the number of needed optimal staff for each year by staff type are presented in Annex 1.

**Table 2:** number of available, required and optimal staff for first year (2020/2021)

Staff Type	% of time dedicated to treating HIV patients	Baseline			Scenario		
		No of Available* Staff	No of Required Minimum* Staff	No of Optimal* Staff	No of Available* Staff	No of Required Minimum* Staff	No of Optimal* Staff
**Doctor**	50%	5	4	5	5	5	6
**Medical lab scientist**	50%	1	2	2	1	2	2
**HIV specialist nurse**	50%	2	2	2	2	2	2
**Dietician**	50%	1	1	1	1	1	1
**General nurse**	50%	2	1	2	2	2	2
**Counsellor**	50%	2	2	2	2	2	2

**Available**: The number of available staff; **Required Minimum**: Minimum number of staffs that the service should have at 100% utilisation rate; **Optimal**: The number of staffs the service should have to ensure a maximum of 80% utilisation rate

**Budgeting and financial planning:** the cost of treating PLWH and patients in prevention programmes are calculated for each fiscal year. Unit costs for each cost type, first-year and 5-year results (baseline and scenario) are presented in Table 3. Total costs are expected to increase to $4,239,640 from $3,337,544 after 5 years due to the increase in prevention programme. The difference in the costs associated with prevention programme increased by $915,200, as the drug costs are extremely high (i.e. $800/patient per year). However, the cost of treating PLWH is reduced by $13,104 as a result of a reduction in the number of HIV positive patients (averted cases). Total costs by expense type can be found for each year and scenario in Annex 1.

**Table 3:** cost of HIV treatment

COSTS	Unit Costs (per patient per year)	Baseline (First Year)	Scenario (First Year)	Baseline (5 years)	Scenario (5 years)
**First Line ART Costs**	$113	$121,588	$121,588	$679,085	$673,005
**Second Line ART Costs**	$250	$18,900	$18,900	$117,200	$122,300
**Laboratory Cost**	$35	$40,306	$40,306	$226,744	$225,575
**Overhead & Personnel Cost**	$328	$377,725	$377,725	$2,124,915	$2,113,960
**PrEP/PEP Costs**	$800	$34,400	$200,000	$189,600	$1,104,800
**TOTAL COSTS**		**$592,919**	**$758,519**	**$3,337,544**	**$4,239,640**

## Discussion

The outcome of our simulation model revealed the beneficial impact of the prevention measures on the HIV programme in Nigeria. A scenario-based on projections from increasing the number of HIV patients currently on preventive therapy (PrEP/PEP) at the facility from 43 to 250 with a 50/50 split. Our study currently showed low uptake in PrEP/PEP programme. However, given some improvement in preventive care, there will be increase in the averted HIV cases, remarkable reduction in service demand and utilization but increased requirement in human resource and financial burden.

Despite the evidence in the literature on the efficacy of PrEP/PEP in the reduction of HIV transmission, the uptake has been particularly low in the study centre. This is similar to the slow initial uptake of PrEP in surveys in the US among men who have sex with men (MSM) where only a minimal proportion (1-3%) of the eligible respondents had taken PrEP in the first year of its introduction [17, 18]. Recently, the studied centre commenced PrEP programme following the national directive on its initiation in at-risk individuals, particularly among serodiscordant couples in the country. This preventive measure is gradually being introduced in HIV negative spouses who were ready to adhere to the treatment and other complementary measures to prevent sexual transmission of HIV. On the other hand, the few cases of PEP uptake despite the awareness and availability of antiretroviral medications could reflect the effective precautionary measures against occupational exposure to HIV infections among health professionals. Furthermore, the stigma associated with rape in our society could further prevent early presentation of victims for non-occupational PEP and the time lag of 72 hours might have been exceeded at the period of medical consultation.

The projected improvement in the preventive care, over the next five years, was related to the increase in the averted HIV cases which as expected would reduce the disease transmission and ultimately decrease in the reported HIV cases. Baeten *et al*. reported 66% and 84% efficacy in men and women respectively who were among the 4,758 serodiscordant heterosexual couples in a PrEP study [19]. Likewise, PrEP has been found to be highly effective in serodiscordant in the Botswana study [20]. Similar to the PrEP regimen, where the concerned individual must seek healthcare and adhere strictly to the chemoprophylaxis, PEP must also commence treatment within 72 hours of exposure for efficacy of the treatment.

With the potentials of PrEP/PEP administration as a comprehensive HIV preventive measure, which would also reduce the demand and utilization of the health facility following a decrease in the new HIV infections. The HIV Prevention Trials Network (HTPN) 052 trial buttressed the fact that early HIV antiretroviral treatment (ART) prevents HIV transmission to the uninfected partner in heterosexual HIV-discordant couples [21]. This would probably prevent the uninfected partner from actively utilizing the HIV treatment facility and remarkably reduce the service demand and utilization. Ultimately, this would reduce the strain on the healthcare system which is already overburdened with several communicable and non-communicable diseases against limited resources.

Furthermore, the effectiveness of these preventive measures would depend on the uptake of HIV in the uninfected partner who would intermittently access a health facility for regular testing to ascertain serostatus and refill on antiretroviral drugs. This would slightly increase the human resource requirement of the hospital, although, not to the extent of having both partners being HIV positive. This projection is similar to the findings by Bor *et al*.at the onset of the HIV/AIDS pandemic in the United Kingdom, where an increase in the HIV/AIDS workload was experienced in the HIV treatment facility. It was attributed to the rising incidence of HIV/AIDS in the community, increase in the new referrals, and subsequent follow-up visits [22].

The President´s Emergency Plan for AIDS Relief (PEPFAR) funding programme has over the years partnered with the Nigerian government in the provision of the required treatment for HIV patients at no cost to the patients. This treatment intervention remains dependent on external donor funding and yet to transition into a complete country-owned programme. With the dwindling funding by the partners, the recently introduced HIV prevention programme- PrEP is yet to achieve full coverage in most HIV treatment centres in the country. The simulation estimated an increase in the total cost over the next five years due to the expected increase in the number of recruited individuals in the prevention programme. Ultimately, the immediate financial burden will be upturned by the long-term gains of breaking the chain of HIV transmission through prevention and averted cases. In a study conducted in Thailand, it was more cost-effective to provide PrEP solely for high-risk individuals within a subgroup of at-risk patients. This cost-effectiveness was however identified as mixed with several confounding variables like unit cost of PrEP, peculiarity of the health facility and the key population involved [23].

The possible limitation to this simulation is the challenge with the adherence to antiretroviral medications especially among the PrEP group who are likely to have misgivings about using medication for a longer period, and the resulting loss of motivations to continue on regular visit to the clinic. This could be prevented by ensuring only highly motivated individuals are recruited and they are constantly given moral incentives to the disease progression in their spouse and the risk reduction of HIV transmission.

The WISN methodology factors in the possible limitations in the execution of clinical and administrative roles in staff employment, such as sickness leave, annual leave, public holidays, meetings, training, etc. Furthermore, we also considered that staff may not always be attending to only HIV patients and, in most cases, they are also responsible for or involved in the management of non-HIV patients too.

## Conclusion

An effective implementation of PrEP/PEP programme in HIV management offers an additional safety approach to break the cycle of HIV transmission especially in the high-risk group and the possibility of ending HIV epidemics. The tool deployed would assist to understand the impacts of scaling up or down, HIV prevention programmes and to test the impacts on the available resources within the HIV treatment pathways. This can be used within a single centre or national level for planning and decision-making purposes. This is important and urgently needed when rolling out prevention programmes as part of the efforts to combat the public health burden of HIV/AIDS.

### What is known about this topic

- Globally, HIV/AIDS is still a burden to the healthcare system and a major public health challenge;
- The effective implementation of PrEP/PEP program offers an additional safety measure to prevent disease transmission in at-risk patients and reduce the overall HIV burden;
- One of the barriers to implementing prevention programs is the lack of a tool to understand the knock-on effect on monitoring visits, staff workload, capacity and the potential number of averted cases.

### What this study adds

- Our application demonstrates the potential and impacts of PrEP/PEP on the prevention of viral transmission in the HIV treatment pathway at the HIV service;
- The impact of increasing prescription of PrEP/PEP for people at risk of HIV transmission would improve budgeting and financial planning by policymakers in the HIV programme;
- The application will assist in human resource management for efficiency in the HIV treatment pathway and decision-making.

## Annex

**Annex 1**: simulation outputs per year
